# Clinical advantage of multidetector computed tomography‐guided trans‐septal puncture

**DOI:** 10.1002/joa3.13004

**Published:** 2024-02-05

**Authors:** Naoya Kataoka, Teruhiko Imamura

**Affiliations:** ^1^ Second Department of Internal Medicine University of Toyama Toyama Japan

To editor,

Lu et al. conducted a comparative analysis between the applicability of multidetector computed tomography (MDCT)‐guided trans‐septal puncture (TSP) and intra‐cardiac echocardiography (ICE)‐guided TSP in the context of catheter ablation for atrial fibrillation.^1^ Their study illustrated that cardiac MDCT offered enhanced visualization of the interatrial septum (IAS) orientation, allowing for precise identification of the appropriate right anterior oblique angle for TSP. Their findings suggested that MDCT‐guided TSP might parallel the feasibility achieved with ICE guidance. However, several pertinent concerns have emerged.

Prior to the integration of ICE, contrast radiography served as our modality for guiding TSP. Although the authors advocate for the pre‐procedural use of MDCT with contrast agents in catheter ablation,^1^ the indispensable reliance on contrast agents remains unresolved, thus casting doubt on the distinct advantages of MDCT guidance.

The disparity in pressure between the left and right atria results in the convexity of the IAS towards the right atrium. Consequently, presuming the IAS to exhibit linearity, as suggested by the authors, may pose challenges.

Beyond the puncture angle, the proximity of the puncture site to the posterior wall of the left atrium emerges as another critical concern when advancing the SL‐0 sheath towards the left atrium. A shorter distance increases the risk of left atrial injury from the advancing SL‐0 sheath. The assessment of this distance without the aid of ICE guidance presents a challenging task.

Conversely, modern puncture systems, such as the radiofrequency needle combined with a rigid pigtail trans‐septal system, obviate the necessity for sheath advancement and may offer a safer alternative compared to conventional methods.^2^

## CONFLICT OF INTEREST STATEMENT

Authors declare no conflict of interests for this article.
